# Materiality and Cognitive Development: Contemporary Debates and Empirical Studies in Early Childhood

**DOI:** 10.5964/ejop.14433

**Published:** 2024-05-29

**Authors:** Nicolás Alessandroni, Juliene Madureira Ferreira

**Affiliations:** 1 Department of Psychology, Concordia University, Montreal, Canada; 2 Faculty of Education and Culture, Tampere University, Tampere, Finland

Developmental psychology has a rich legacy of contributions challenging mainstream, Cartesian-rooted perspectives of cognition. Indeed, pioneering figures within the field championed non-reductionist, anti-dualistic, and anti-representationalist views of cognitive development closely aligned with contemporary perspectives. Gesell, a prominent example, advocated for a monistic, functional perspective focused on studying meaningful behaviour. He famously proposed that the “mind” is the manifestation of characteristic behavioural patterns, emphasizing that research should elucidate how the morphology of behaviour develops over time (Gesell, 1935, 1938):

Behaviour has shape. The shapes which behaviours assume can be investigated in their own scientific right. A morphological approach leads to the description and measurement of specific forms; the systematic study of topographic relations and correlations of such forms, their ontogenetic progression (Gesell, 1938, p. 229).

In a related vein, the work of E. J. Gibson in ecological psychology consistently emphasized the critical role of affordances in understanding behaviour within the dynamic interplay of individual and environment, profoundly shaping cognitive development. In a noteworthy article, Gibson articulated a research program for developmental psychology organized around what she identified as the *five hallmarks of human behaviour*—*agency*, *prospectivity*, *flexibility*, *communicative creativity*, and *retrospectivity*. Importantly, she argued that these hallmarks could all be investigated by describing the behaviour of an organism functioning adaptively in a “world of events and places and people” while analyzing “the development of this activity with insight into the constraints, opportunities, and environmental offerings that underlie the dynamics of change” (E. J. Gibson, 1994, p. 71).

Recent scholarly literature on *5E cognition* (i.e., *embodied*, *enactive*, *extended*, *embedded*, and *ecological*) has sparked renewed interest in approaches similar to those of Gesell and Gibson. Within this literature, cognition is conceptualized in terms of dynamic interactions between agents and their environments (Gallagher, 2020; Kiverstein & Rietveld, 2021; Newen et al., 2018), while cognitive development is viewed as the incremental enaction of practical skills (e.g., Nonaka & Goldfield, 2018; Travieso et al., 2020; Yakhlef & Rietveld, 2020), habits (e.g., Brinck & Reddy, 2020; Sheets-Johnstone, 2014), recurrent forms of material engagement (e.g., Alessandroni, 2021, 2023; Aston, 2020, 2022; Malafouris, 2019, 2020; Prezioso & Alessandroni, 2023), as well as expectations, anticipations, coordinations, and potentials for behaviour that unfold across various timescales (e.g., Adolph, 2019; Ossmy & Adolph, 2020).

In a thought-provoking editorial featured in this journal in 2015, C. Moro rightly pointed out that, by and large, psychology has neglected to explore how materiality relates to cognition. Despite psychology's increasing acknowledgment of cultural factors, material culture seems to have persisted as “terra incognita” (Moro, 2015, p. 172). In underscoring the drawbacks of this neglect, Moro contended that “material culture and material objects are at the heart of human developmental processes, conditioning our everyday relationship to the world, and influencing how we live, act, think and develop as human beings from an early age” (Moro, 2015, p. 172), and welcomed the increasing stream of interdisciplinary efforts focused on materiality and cognition. The increasing traction gained by 5E perspectives in recent years has significantly advanced our understanding of how materiality shapes human development, fostering new opportunities for (inter)action across multiple timescales and social contexts. This progress is exemplified by contributions stemming or gaining insights from frameworks such as Material Engagement Theory (e.g., Alessandroni & Malafouris, 2023; Barona, 2021; Gallagher & Ransom, 2016; Gubenko & Houssemand, 2022; Malafouris, 2013; Malafouris et al., 2023; March & Malafouris, 2023; Paolucci, 2021; Vietri et al., 2023).

Further understanding of how materiality catalyzes cognitive development is particularly relevant in early childhood education, where the foundations for several cognitive processes are laid. As the *common worlds pedagogy* theory posits, children, teachers, and objects are *equally active participants* in creating the dynamics of the expanded community they form. Within this community, objects:

(...) beckon and pull us in. They live, speak, gesture, and call to us. Materials can evoke memories, narrate stories, invite actions, and communicate ideas (...) Thinking with materials transforms early childhood education, provoking educators to notice how materials and young children live entangled lives in classrooms, how they change each other through their mutual encounters (Pacini-Ketchabaw et al., 2016, pp. 1–2).

In line with the ever-growing interest in materiality and cognition within psychology and beyond, this special issue features an assembly of five theoretical and empirical contributions ultimately aimed at unravelling and better understanding how interactions between children, adults, and objects shape cognitive development in early childhood and the potential consequences this may have for early childhood education.

In “An Ecological Approach to Conceptual Thinking in Material Engagement,” Nicolás Alessandroni, Lambros Malafouris, and Shaun Gallagher explore the process and meaning of conceptual thinking through the lens of Material Engagement Theory. Building upon the notion of *thinking as thinging*, the authors offer a fresh perspective that challenges mainstream representationalist views. Specifically, Alessandroni et al. contend that conceptual thinking is not an abstract activity involving mental representations *of* the world but rather a kind of unmediated practical knowledge that individuals put into play when they engage, in a general way, *with* and *through* the world. In this sense, they propose that conceptual thinking is instantiated in the dynamic coordination of bodily practices and artifacts within sociomaterial activities. Significantly, the authors introduce seven clear principles for defining conceptual thinking within a post-cognitivist, ecological-enactive framework of cognition, thus furthering the field of 5E cognition studies and opening up new avenues for empirical exploration.

“Babies in the Corporeal Turn: The Cognitive Embodiment of Early Motor Development and Exploration in the Brazilian Context of Early Childhood Education” by Natalia Meireles Santos da Costa, Joana de Jesus Andrade, and Aline Patrícia Campos Tolentino de Lima is an empirical study investigating the motor and cognitive development of babies from an embodied perspective, focusing on the everyday material and sociocultural landscapes where infants develop. The authors highlight the importance of experience and perceptual-motor mechanisms in modulating the development of person-environment systems. They also reflect on the mediating role of public policies and pedagogical intentionality in providing meaning and direction to material compositions and engagement with objects and spaces in educational environments. This macro-to-micro view of the relationship between materiality and children’s motor and cognitive development is particularly relevant to practitioners in Early Childhood Education.

Valentina Fantasia, Francesca Moncalli, and Arianna Bello authored “Shared Construction of Social Pretend Play Sequences in Kindergarten,” which delves into children’s social interactions, engagement with available objects, and the spontaneous creation of pretend play scenarios in kindergarten settings. This study addresses a gap in research regarding the emergence and development of social pretend play in ecological settings before the age of three. Through detailed microanalytical observations of pretend play episodes, the authors illuminate how shared pretend play interactions evolve among young children in a kindergarten environment, influenced by the materiality available for children during play. The research demonstrates that children dynamically manage their participation in play by using local embodied and material resources, as well as sequential action turns. This article addresses the interests of educational psychologists and developmental researchers concerned with the role of materiality in shaping specific social abilities, such as symbolic play.

Relatedly, the study “Gestures, Objects, and Space: Exploring Teachers’ Multimodal Communication in Nursery Schools” by Ana Moreno-Núñez and Marta Casla examines the relationship between materiality and teachers’ communicative strategies during group interactions in nursery schools with two- to three-year-old children. Of particular interest to this special issue is Moreno-Núñez and Casla’s view that materiality and the classroom layout foster children’s and adults’ possibilities for experimentation and participation in sensorimotor experiences that lead to meaning-construction processes. By investigating the multimodality and materiality involved in adult-child communication in a naturalistic context, this study brings new and important insights into the extent to which the use of objects and space shape the linguistic contexts of young children and the potential opportunities and limitations they pose for classroom interactions.

The fourth empirical article—by Juliene Madureira Ferreira and Luciana Soares Muniz—is entitled “Material Engagement Shaping Participation of Children on the Autism Spectrum: Embodiment and Subjectivity in Small-Group Learning.” This article reports a study investigating how materiality influences possibilities for participation in small-group learning in special education settings, specifically focusing on children on the Autism Spectrum. The authors introduce a specific pedagogical practice: the *idea diary*. This practice not only enriches the possibilities for children on the spectrum to share their worlds and learning interests with their typically developing peers—thus expanding children’s engagement with learning situations—but also serves as a window into children’s subjectivity. The idea diary transcends being a channel for children’s expression and communication of their subjective meanings. In fact, by the affordances it facilitates, the materiality of the idea diary fosters a unique form of (inter)subjectivity that would not exist otherwise. This study provides empirical support to the argument that material engagement enables specific forms of interaction and participatory sense-making in group learning situations.

In closing, we invite readers to explore the diverse contributions within this special issue. These studies provide both compelling theoretical arguments and empirical data regarding the vital role of material culture in human development. By highlighting its potential to spur cognition and interaction across timescales and social settings, the reported findings contribute to the contemporary understanding of how materiality shapes human experiences and behaviours.
